# Pyrrolidine Dithiocarbamate Ameliorates Sepsis-Associated Encephalopathy by Inhibiting Autophagy and Inflammation in the Brain

**DOI:** 10.1007/s11064-025-04355-5

**Published:** 2025-02-27

**Authors:** Yang Lu, Zhiyi Zuo

**Affiliations:** 1 Department of Anesthesiology, University of Virginia, Charlottesville, VA 22908 USA; 2 Department of Anesthesiology, Second Affiliated Hospital, Xi’an Jiaotong University, Xi’an, Shaanxi 710004 China; 3 Department of Anesthesiology, University of Virginia Health System, 1 Hospital Drive, PO Box 800710, Charlottesville, VA 22908-0710 USA

**Keywords:** Autophagy, Encephalopathy, Neuronal microstructures, Neuroinflammation, Sepsis

## Abstract

Sepsis-associated encephalopathy (SAE) is common and has poor clinical outcome. Sepsis increases autophagy in the brain. This study was designed to determine the role of autophagy on SAE including the brain structures related to learning and memory and the effects of pyrrolidine dithiocarbamate (PDTC), an anti-inflammatory agent, on autophagy and SAE. Six- to eight-week old CD-1 male mice were subjected to cecal ligation and puncture (CLP). Some mice received intracerebroventricular injection of the autophagy suppressor 3-methyladenine (3-MA) or intraperitoneal injection of PDTC immediately at the completion of the CLP. ELISA was used to measure interleukin (IL)-1β, IL-6, IL-10, and tumor necrosis factor α. Autophagy-related protein expression in the cerebral cortex and hippocampus was analyzed by Western blotting. The cognitive functions of mice were analyzed by Barnes maze and fear conditioning tests. CLP increased microtubuleassociated protein light chain 3 II (LC3II) and Beclin 1 and decreased p62 in the brain. CLP also increased proinflammatory cytokines and impaired learning and memory. These effects were inhibited by 3-MA and PDTC. Spine proliferation and maturation were impaired by CLP, which was attenuated by PDTC and 3MA. Abundant autophagic vacuoles were observed by transmission electron microscopy in CLP group. LC3II immunostaining was co-localized with that of ionized calcium-binding adapter molecule 1 and microtubule-associated protein-2. The co-staining was attenuated by 3-MA and PDTC. Our results suggest that sepsis increases autophagy in the microglia and neurons. Inhibiting autophagy improves SAE and brain structures related to learning and memory in mice. Autophagy and inflammation in the brain may regulate each other during sepsis.

## Introduction

Sepsis is a complex and frequently fatal condition. As a leading cause of mortality in intensive care units, sepsis requires a lot of health care resources. However, its morbidity remains high [1–3]. It is recognized that exaggerated systemic inflammatory responses and disrupted immune suppression caused by sepsis are the main factors to affect the outcome of the patients [3, 4]. One of the common conditions in patients with sepsis is sepsis-associated encephalopathy (SAE). SAE presents with neurocognitive dysfunction [5, 6]. The role of inflammation in SAE has been demonstrated [7, 8]. Recent studies have indicated a role of autophagy in SAE [9–11].

Autophagy is a process to remove intracellular unnecessary materials or damaged components, including proteins and organelles for recycling [12, 13]. As an evolutionarily conserved system, autophagy is vital for cell survival and intracellular homeostasis. The process of autophagy has three main steps, autophagosome formation, autophagosome lysosome fusion, and the degradation of the autophagosome contents delivered to lysosome [14–16]. Studies have shown an increase in autophagic vacuoles and autophagy-associated proteins in septic animals [17]. For instance, the expression of microtubule-associated protein light chain 3 (LC3), a marker for the formation of an autophagosome, is increased 6 h after cecal ligation and puncture (CLP) [18]. It is indicated that the increased autophagy is protective against SAE [9, 11, 19], although different findings have also been reported [10]. A very recent study has shown that inhibiting autophagy attenuates lipopolysaccharide-induced increase of inflammatory cytokines and neuronal injury in the hippocampus [20]. Interestingly, autophagy has been known to regulate inflammatory response [21, 22]. For example, autophagy can regulate the microglial activation, although inhibiting inflammation is reported to be achieved by increasing and decreasing autophagy in microglia in different studies (reviewed in [23]). Similarly, inflammation can regulate autophagy via its component molecules. For example, inflammatory cytokines induce autophagy (reviewed in [24]). However, the interaction between autophagy and inflammation in the context of SAE is not clear.

This study is designed to determine the role of autophagy in SAE and the interaction between autophagy and inflammation in the brain of individuals with sepsis. For this purpose, mice were subjected to CLP in the presence or absence of pyrrolidine dithiocarbamate (PDTC), a small molecule with potent anti-oxidant and anti-inflammatory property under various conditions [25, 26], or 3-methyladenine (3-MA), an autophagy inhibitor [27]. CLP is a commonly used sepsis model that shares many features of human sepsis [28]. The learning, memory, autophagy, and inflammation in the brain were assessed.

## Materials and Methods

### Animals

All animal protocols were approved by the Institutional Animal Care and Use Committee of the University of Virginia (Charlottesville, VA, USA). The approved protocol number was 3114. All surgical and experimental procedures were carried out in accordance with the National Institutes of Health Guide for the Care and Use of Laboratory Animals (NIH publications number 23–80) revised in 2011. All mice were kept in a vivarium room at constant temperature (23 ± 2℃) and 12 h light/dark cycle with free access to food and water. Six- to eight-week-old male CD-1 mice were purchased from Charles River (Wilmington, MA). Our manuscript was written up in accordance with the Animal Research: Reporting In Vivo Experiments.

### Animal Groups

CLP successfully induced SAE in mice in our previous study [7]. This current study was designed to determine the role of autophagy and inflammation in SAE. For this purpose, CD-1 mice were randomly assigned to four groups: sham-operated group, CLP group, 3-methyladenine (3-MA) group, and PDTC group. The 3-MA and PDTC groups had CLP in addition to receiving 3-MA and PDTC treatments, respectively. A control group that animals did not have any surgery or treatments was not included because the sham-operated group was not different from the control group in the inflammatory cytokine levels, learning and memory in our previous study [7]. Control groups that animals with CLP received injection of solvent (normal saline, NS) for 3-MA and PDTC were not included because the injections of solvents alone into the brain did not affect the presentations of SAE [7]. Limiting these non-essential groups simplified our study. The learning and memory of the 4 groups of mice were assessed by Barnes maze and fear conditioning tests from 6 days after the CLP. Different cohorts of mice with the same experimental conditions were sacrificed at 24 h after surgery. Their brains were harvested for Western blotting, immunohistochemistry, ELISA enzyme-linked immunosorbent assay (ELISA), Golgi staining, and electron microscopy. The sample size per experimental group was from 7 to 14 depending on the tests of experiments (more animals per group were used in behavioral studies).

### Anesthesia and Surgery

Polymicrobial sepsis was induced by CLP with a method as we described previously [7]. Animals were anesthetized with 1.8% isoflurane. A 1-cm long ventral midline incision was performed. The cecum was then carefully exposed, ligated just distal to the ileocecal valve with a 3–0 silk suture to avoid intestinal obstruction, and punctured twice using a 19-gauge needle. The punctured cecum was squeezed to expel a small amount of fecal material and returned to the abdominal cavity. The abdominal incision was closed in layers with a 7 − 0 silk suture and wound clips. The wound was infiltrated with 0.25% bupivacaine. Immediately after the surgery, some mice received an intracerebroventricular injection of the autophagy suppressor 3-MA (200 nmol in NS, Sigma, M9281) or intraperitoneal injection of PDTC (50 mg/kg in NS, Sigma, P8765), an anti-inflammatory agent [25, 26]. All animals received subcutaneous administration of 1 ml NS immediately after the operation to provide fluids. Sham-operated mice had the abdominal incision but did not have cecum ligation and puncture.

### Barnes Maze Test

Six days after surgery, mice were assessed by Barnes maze test as we described before [7]. The Barnes maze (SD Instruments, San Diego, CA) is a circular platform with 20 equally spaced holes. One of the holes was connected to a dark chamber that was called target box. The test was started by placing mice in the center of platform. Aversive noise (85 dB) and bright light (200 W) shed on the platform were used to encourage mice to find the target box. They were trained in 4 consecutive days with 3 min per trial, 2 trials per day and 15 min between trials. Their reference memory was then tested on day 5 (short-term retention) and day 12 (long-term retention). Each mouse had one trial on each of these two days. No test was performed during the period from day 5 to day 12. The latency to find the target box during each trial was recorded with the assistance of ANY-Maze video tracking system (SD Instruments).

### Fear Conditioning Test

Mice were assessed by fear conditioning test 24 h after the Barnes maze test. Each mouse was placed in a test chamber wiped with 70% alcohol and subjected to three tone-foot shock pairings (tone: 2000 Hz, 85 db, 30 s; foot shock: 0.7 mA, 2 s) with a 1-min interval between pairings in a dark room. The mouse was removed from the test chamber after training. The mouse was placed back to the same chamber 20 h later for 6 min without receiving tone or shock stimulation. The amount of time with freezing behavior was recorded in the 6-min interval (context-related freezing behavior). The mouse was then placed in a different test chamber wiped with lemon juice 2 h later in a light room. After 3 min without any stimuli, the tone stimulus was turned on for 30 s followed by 1-min interval for three cycles (4.5 min in total). The freezing behavior in this 4.5-min interval was recorded (tone-related freezing behavior). The time of freezing behavior was counted by an observer who was blind to group assignment of animals.

### Brain Tissue Harvest

Mice were deeply anesthetized with isoflurane and perfused transcardially with NS. The cerebral cortex and hippocampus from Bregma − 2 to -5 were harvested for immunohistochemistry. Also, the cerebral cortex and hippocampus were dissected out immediately after transcardial perfusion for ELISA and Western blotting. These brain tissues were harvested 24 h after surgery. Brain tissues were harvested 7 days after surgery for Golgi staining.

### Western Blotting

Briefly, cerebral cortex and hippocampus tissues were homogenized in RIPA buffer (Sigma-Aldrich, St. Louis, MO) containing protease inhibitor cocktail (10 mg/ml aproteinin, 5 mg/ml pepstatin, 5 mg/ml leupeptin, and 1 mM phenylmethanesulfonylfluoride) and placed on ice for 30 min. The homogenates were centrifuged at 13,000 rpm for 25 min at 4 °C. The supernatant was collected for Western blotting. Protein concentration was determined by BCA assay.

Twenty micrograms of proteins per lane were separated on a polyacrylamide gel (Catalog number: 456–1025; Bio-rad, Hercules, CA) and then blotted onto a polyvinylidene difluoride membrane. The membranes were blocked with Protein-Free T20 Blocking Buffer (Catalog number: 37573, Thermo Scientific, Logan, UT) and incubated with the following primary antibodies overnight at 4 °C: the rabbit polyclonal anti-LC3B antibody (1:500 dilution; catalog number: 48394; Abcam), the rabbit polyclonal anti-p62 antibody (1:500 dilution; catalog number: 56416; Abcam), the rabbit polyclonal anti-Beclin-1 antibody (1:500 dilution; catalog number: 62557; Abcam), and the rabbit polyclonal anti-glyceraldehyde 3-phosphate dehydrogenase (GAPDH) antibody (1:2000 dilution; catalog number: G9545; Sigma-Aldrich). Appropriate secondary antibodies were used. Protein bands were visualized by Genesnap version 7.08 and quantified by Genetools version 4.01. The relative protein expression was normalized to those of GAPDH proteins from the same sample to control for errors in protein sample loading and transferring during Western blotting analysis, respectively. The results from animals under various experimental conditions were then normalized by the mean values of the corresponding control animals.

### Immunohistochemistry

Cerebral hemisphere from Bregma − 2 to -5 was harvested, fixed in 4% paraformaldehyde in 0.1 M phosphate-buffered saline at 4 °C for 24 h, and embedded in paraffin. Coronal sections at 5 μm were cut and mounted on slides. Antigen retrieval was performed in sodium citrate buffer (10 mM sodium citrate, 0.05% Tween 20, pH 6.0) for 20 min. The slides were immersed in 5% normal goat serum and 1% bovine serum albumin (BSA) in Tris-buffered saline plus 0.05% triton-X 100 (TBST) for 2 h at room temperature. The sections were incubated with (1) the rabbit polyclonal anti-LC3B antibody (1:500 dilution; catalog number: 48394; Abcam) and mouse monoclonal anti-microtubule-associated protein-2 (MAP-2) antibody (1:500 dilution, catalogue number: ab254143; Abcam); (2) the rabbit polyclonal anti-LC3B antibody (1:500 dilution; catalog number: 48394; Abcam) and goat polyclonal anti-glial fibrillary acidic protein (GFAP) antibody (1:500 dilution, catalog number: 53554; Abcam); or (3) the rabbit polyclonal anti-LC3B antibody (1:500 dilution; catalog number: 48394; Abcam) and goat polyclonal anti-ionized calcium-binding adapter molecule 1 (Iba-1) antibody (1:500 dilution, catalog number: 5076; Abcam) at 4 °C overnight. Sections were rinsed in TBS. The brain sections were then incubated for 1 h at room temperature in a dark room with (1) the donkey anti-rabbit IgG antibody conjugated with Alexa Fluor 594 (1:200 dilution, catalog number: A-21209; Invitrogen, Eugene, ON) and the donkey anti-mouse IgG antibody conjugated with Alexa Fluor 488 (1:200 dilution, catalog number: A-21202; Invitrogen); (2) the donkey anti-rabbit IgG antibody conjugated with Alexa Fluor 594 (1:200 dilution, catalog number: A-21209; Invitrogen) and the donkey anti-goat IgG antibody conjugated with Alexa Fluor 488 (1:200 dilution, catalog number: A-11055; Invitrogen); or (3) the donkey anti-rabbit IgG antibody conjugated with Alexa Fluor 594 (1:200 dilution, catalog number: A-21209; Invitrogen) and the donkey anti-goat IgG antibody conjugated with Alexa Fluor 488 (1:200 dilution, catalog number: A-11055; Invitrogen). After being washed in TBS, sections were counterstained with DAPI (1:1000 dilution, catalog number: MBD0020; Millipore Sigma) for 5 min, then rinsed and mounted with Vectashield mounting medium (catalog number: H-1000; Vector Laboratories). Images were acquired with a fluorescent microscope with a charge-coupled device camera (Olympus DP70, Olympus Corporation, Tokyo, Japan).

In all immunostaining studies, a negative control omitting the incubation with the primary antibody was included. Quantification was performed as follows. Briefly, six sections per mouse in the cerebral cortex area or hippocampus were randomly acquired, and one independent microscopic field in each section was imaged. The areas in the image with intensity above a predetermined threshold level were considered positively stained areas. This measurement was performed by using Image J 1.47n software. The degree of positive immunoreactivity was reflected by the percentage of the positively stained area in the total area of interested structure in the imaged field. All quantitative analyses were performed in a blinded fashion.

### ELISA Assay of Cytokines

Interleukin (IL)-1β, IL-6, IL-10 and tumor necrosis factor α (TNFα) levels in the cerebral cortex and hippocampus were determined with Quantikine ELISA kits (R&D Systems, Minneapolis, MN) according to the manufacturer’s instructions. Briefly, brain tissues were homogenized on ice in 20 mM Tris-HCl buffer (pH 7.3) for 30 min. Homogenates were centrifuged at 10,000 g for 10 min at 4 °C. The supernatant was ultra-centrifuged at 150,000 g for 2 h at 4 °C. The supernatant was collected for ELISA. The quantity of IL-1β, IL-6, IL-10, and TNFα in each sample was standardized to its protein contents.

### Golgi Staining

Brains were harvested and stained using the FD Rapid Golgi Stain kit (FD Neuro Technologies) [19, 29]. After being immersed in a 1∶1 mixture of FD Solution A∶B for 2 weeks at room temperature in the dark, brains were then transferred to FD Solution C and kept in the dark at 4 °C for 48 h. Solution C was replaced after the first 24 h. Coronal sections of 150 μm thickness were cut with a vibratome (DTK-1500E, DSK Corporation, Pittsburgh) and transferred to gelatin-coated slides (LabScientific) onto small drops of FD Solution C. After allowing sections to dry at room temperature in the dark for at least 4 h or overnight, slides were then stained as described in the FD Rapid Golgi Stain instructions. Permount mounting medium (Fisher) was used for cover-slipping. For each mouse, three independent coronal sections that contained the cerebral cortex and hippocampus (Bregma − 2.5 to − 3.2 mm) were imaged. Secondary and tertiary dendrites of these neurons were selected for analysis. Z-stacks of Golgi-stained dendrites (up to 80 microns in total on Z-axis; optical section thickness at 0.5 μm, i.e., 160 images per stack) were taken at 63x magnification on a Zeiss AxioImager Z2.5. Z-stacks were taken from each mouse. With the freely available RECONSTRUCT software, the images were imported and calibrated before dendritic segment identification and measurement. Spine width measurements made by drawing a straight line across the widest part of the spine head when it was in focus, then scrolling up and down through the Z-stack to get the accurate Z-length of the spines. After the information about the width, length, and length-to-width-ratio (LWR) of the spine was obtained, the spines were classified into different types as the final data. These measurements and analyses were performed as described previously [30, 31].

### Electron Microscope

As we described before [32], the hippocampus for electron microscopy was obtained in a coronal plane (1 mm long) and then fixed in a buffer containing 2% (w/v) paraformaldehyde and 2.5% (w/v) glutaraldehyde in 0.1 M PBS at 4 °C for 24 to 36 h. Afterwards, they were post-fixed in 3% (v/v) glutaraldehyde and 1% (w/v) phosphate-buffered osmium tetroxide and embedded in Epon812. The sections were cut at 0.12 μm thickness and stained with 0.2% (w/v) lead citrate and 1% (w/v) uranyl acetate. The sections were subsequently observed under a JEOL 1230 transmission electron microscope (JEOL, USA).

### Statistical Analysis

Parametric data in normal distribution were presented as mean ± S.E.M. Non-parametric data or parametric data that were not in normal distribution were presented in box plot. Data from the training sessions of Barnes maze were analyzed by a two-way repeated measures analysis of variance followed by Tukey test. The other data were tested by one-way analysis of variance followed by Tukey test or one-way analysis of variance on rank followed by Tukey test as appropriate. Differences were considered significant at *P* < 0.05. All statistical analyses were performed with SigmaStat (Systat Software, Inc., Point Richmond, CA).

## Results

### 3-MA and PDTC Attenuated CLP-Increased Autophagy in the Cerebral Cortex and Hippocampus

**Fig. 1:**
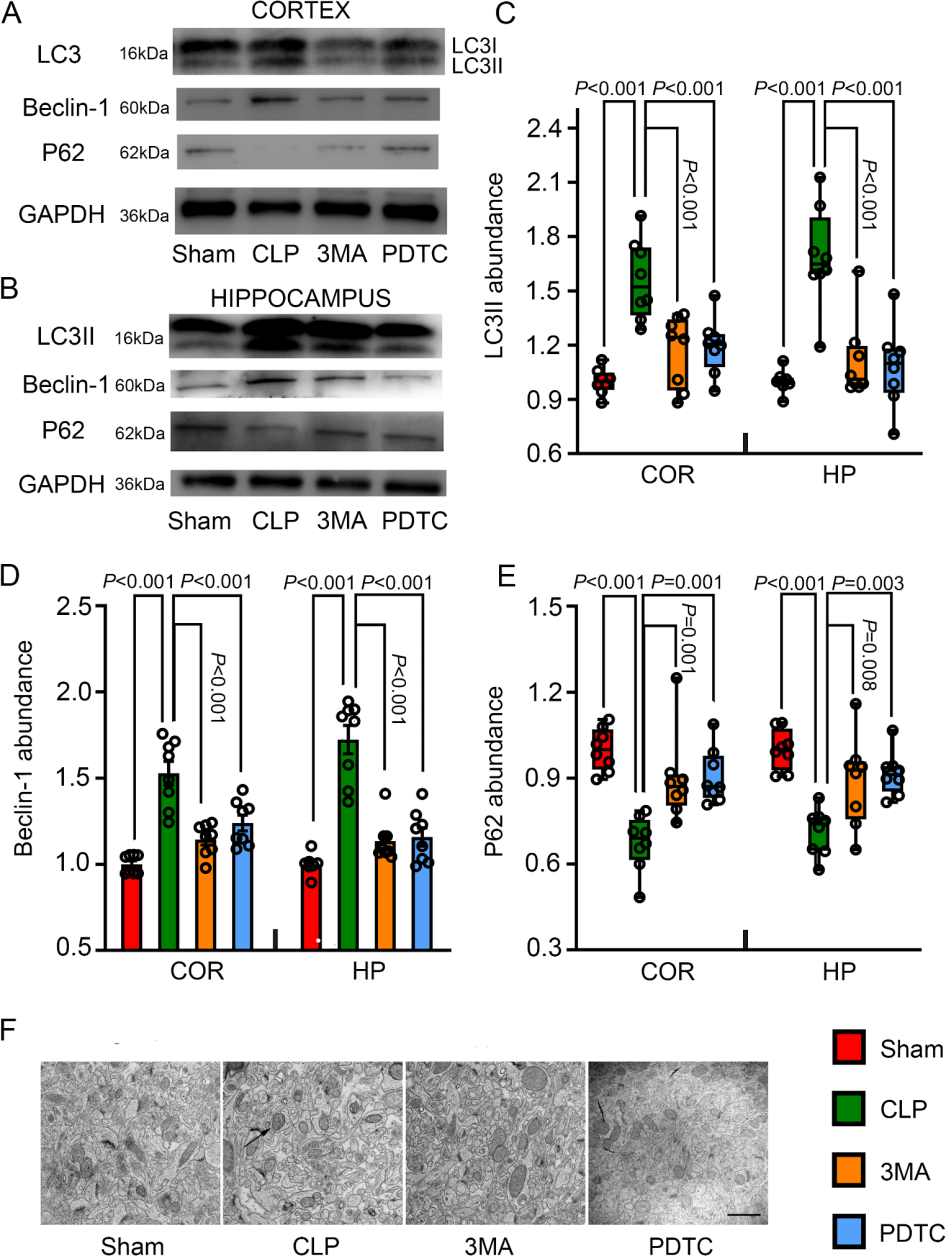
The effects of 3-MA and PDTC on the expression of autophagy-related proteins in mice with CLP. Cerebral cortex or hippocampus was harvested 24 h after CLP. **A**: Representative Western blotting image in the cerebral cortex. **B**: Representative Western blotting image in the hippocampus. **C**: Quantification of LC3II. **D**: Quantification of Beclin-1. **E**: Quantification of p62. **F**: Representative electron microscopic images. Arrow indicates a large autophagosome. Scale bar = 1 μm. Parametric results in normal distribution are in mean ± S.E.M. (panel D) and other results that are not in normal distribution are presented as median with interquartile range (panels C and E). Data of each individual animal is also presented (*n* = 8). COR: cerebral cortex, HP: hippocampus, 3MA: CLP plus 3-MA, PDTC: CLP plus PDTC

Cortical and hippocampal tissues were harvested from mice 24 h after CLP. LC3II was significantly increased in CLP mice compared with that in sham-operated mice (Fig. 1A and C). CLP also increased Beclin-1 and decreased p62 (Fig. 1A, B, D and E). These results suggest that CLP increases autophagy. Consistent with these results, there were autophagosomes containing mitochondria and other organelles and herniation of the outer membranes of endoplasmic reticulum into adjacent lysosomal structures in the hippocampus (Fig. 1F). The effects of CLP on the expression of LC3II, Beclin-1, and p62 were attenuated by 3-MA and PDTC (Fig. 1A and E), suggesting that 3-MA and PDTC attenuate autophagy in the brain of mice with CLP.

### 3-MA and PDTC Attenuated CLP-Increased TNFα and IL-6 in the Brain Tissues

**Fig. 2:**
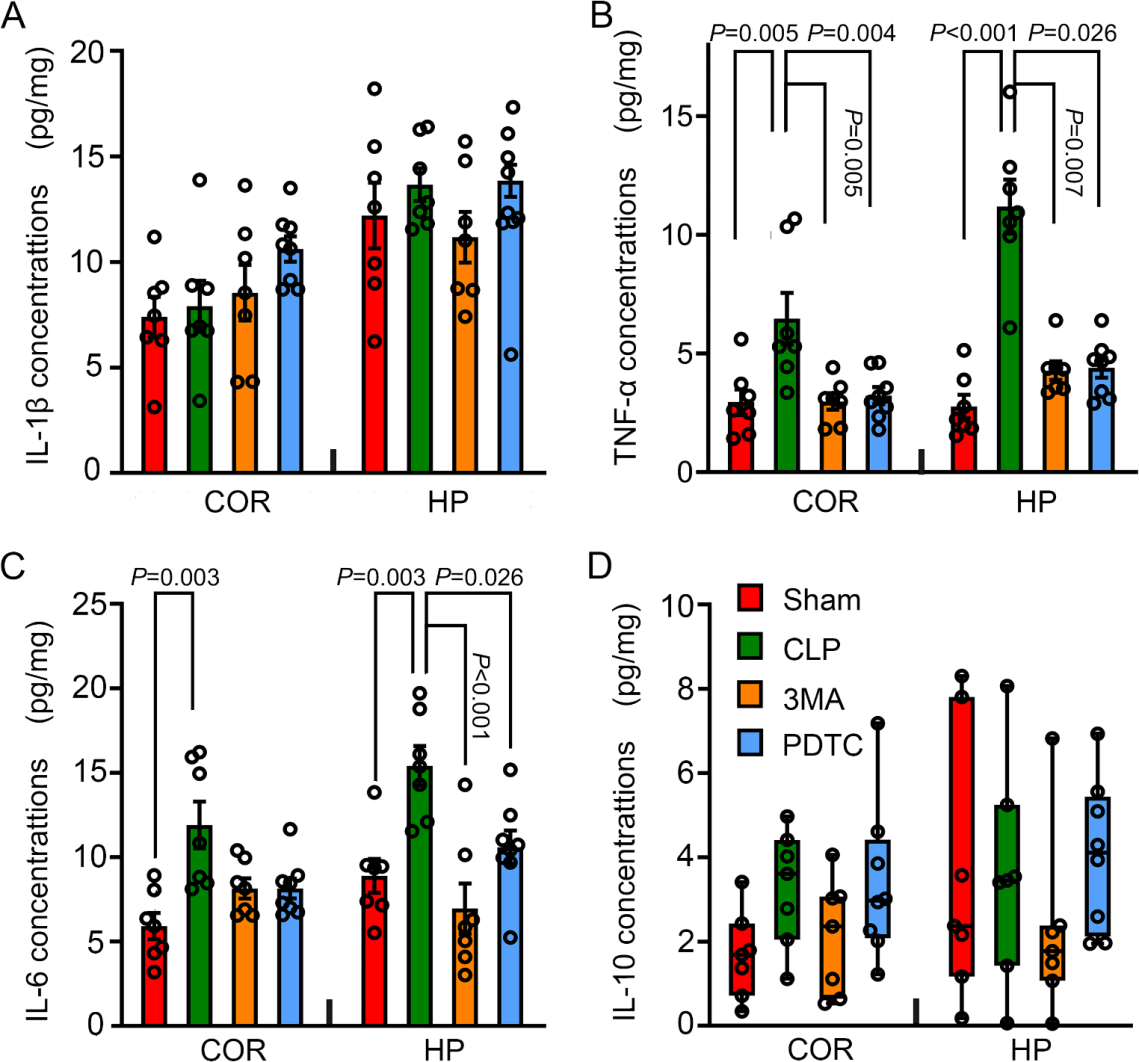
The effects of 3-MA and PDTC on cytokine expression in mice with CLP. Cerebral cortex or hippocampus was harvested 24 h after CLP. **A**: IL-1β concentrations. **B**: TNFα concentrations. **C**: IL-6 concentrations. **D**. IL-10 concentrations. Parametric results in normal distribution are in mean ± S.E.M. (panels A to C) and results in panel D are not in normal distribution and are presented as median with interquartile range. Data of each individual animal is also presented (*n* = 7). COR: cerebral cortex, HP: hippocampus, 3MA: CLP plus 3-MA, PDTC: CLP plus PDTC

There was no difference in IL-1β concentrations in the cerebral cortex and hippocampus of the 4 groups of mice at 24 h after the CLP (Fig. 2A). However, CLP increased the concentration of TNFα and IL-6 in both cerebral cortex and hippocampus, and this increase was attenuated by 3-MA and PDTC (Fig. 2B and C). The expression of IL-10 in the brain tissues was not different among the four groups (Fig. 2D).

### 3-MA and PDTC Attenuated CLP-Induced Learning and Memory Impairment

**Fig. 3:**
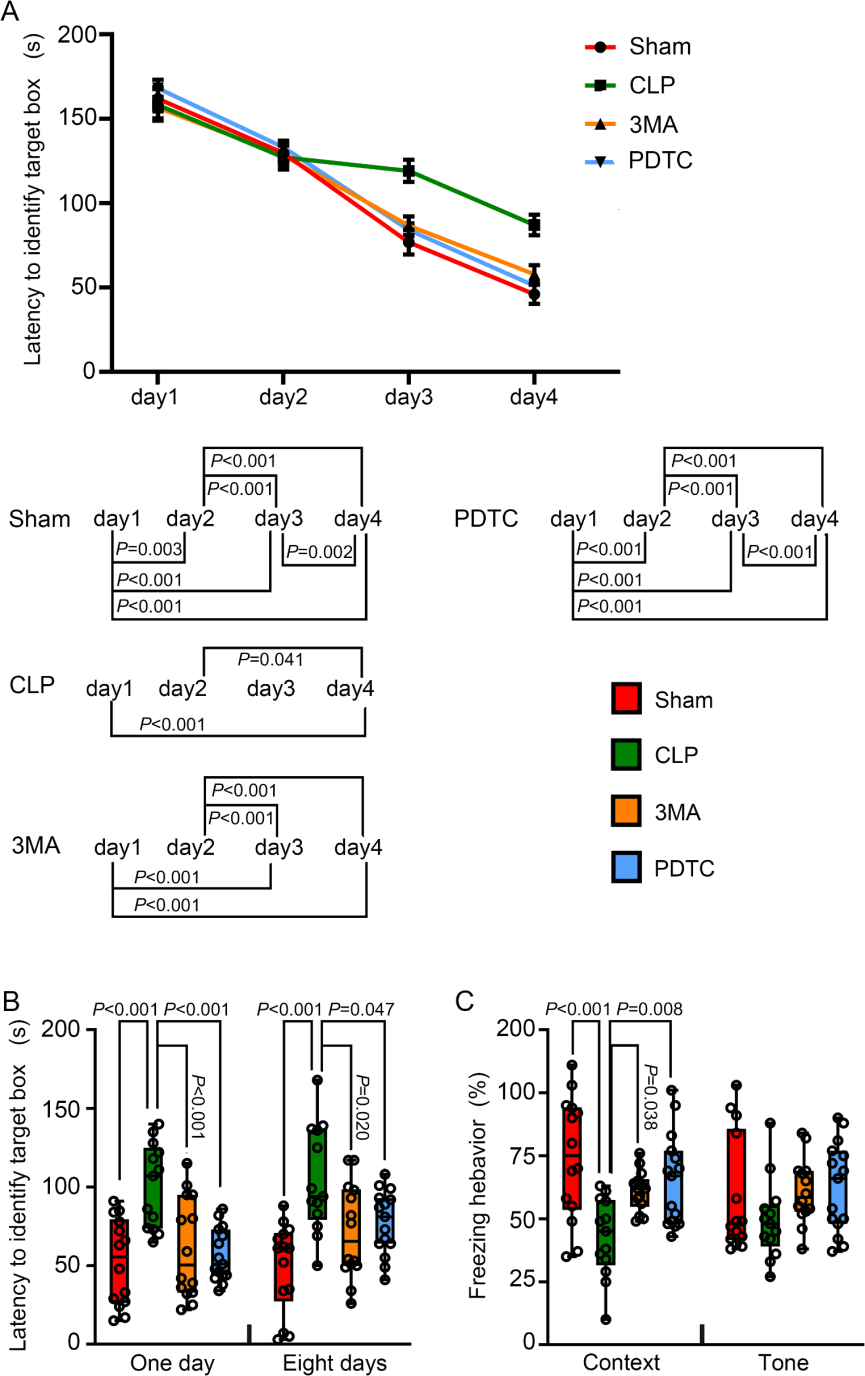
The effects of 3-MA and PDTC on learning and memory of mice with CLP. Mice were subjected to Barnes maze and fear conditioning tests from 1 week after CLP. **A**: Training sessions of Barnes maze test. Top panel: data presented in line plot. Bottom panel: statistical analysis results for the comparisons among different training days within one group of mice. **B**: Memory phase of Barnes maze test. **C**: Context- and tone-related fear conditioning test. Parametric results in normal distribution are in mean ± S.E.M. (panel A) and other results that are not in normal distribution are presented as median with interquartile range (panels B and C). Data of each individual animal is also presented (*n* = 14). COR: cerebral cortex, HP: hippocampus, 3MA: CLP plus 3-MA, PDTC: CLP plus PDTC

The time needed for mice to identify the target box was decreased with increased training sessions in the Barnes maze test. The time needed for performing this task was shorter on day 4 compared with that on day 1 in all 4 groups (Fig. 3A). CLP had a major effect on the time to identify the target box during training sessions [F(1,25) = 10.068, *P* = 0.004). 3-MA and PDTC attenuated this effect of CLP [F(1,25) = 6.660, *P* = 0.016 for 3-MA; F(1,26) = 7.662, *P* = 0.010 for PDTC]. 3-MA and PDTC also attenuated the increased time needed for mice with CLP to identify the target box one day and 8 days after the training sessions in Barnes maze test (Fig. 3B). Similarly, mice with CLP had less context-related freezing behavior than control mice in the fear conditioning test. This decrease was attenuated by 3-MA and PDTC. There was no difference in tone-related freezing behavior (Fig. 3C). These results suggest that CLP impairs learning and memory. This impairment is attenuated by 3-MA and PDTC.

### 3-MA and PDTC Attenuated CLP-Induced Impairment of the Spine Formation and Maturation in Brain Tissues

**Fig. 4:**
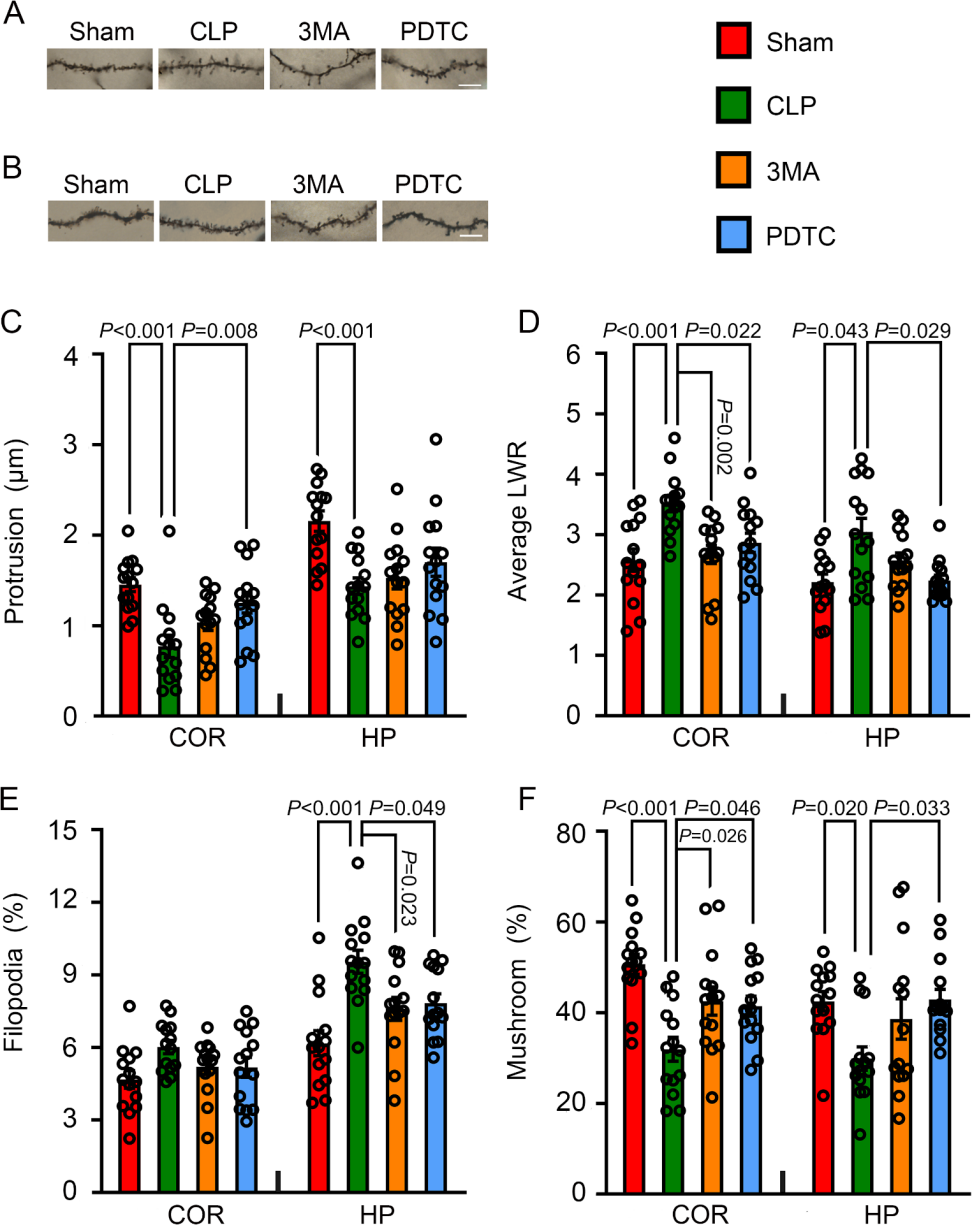
The effects of 3-MA and PDTC on spine formation and maturation in mice with CLP. Cerebral cortex or hippocampus was harvested 7 days after CLP. **A**: Representative images of dendritic spines of cerebral cortex after Golgi-Cox staining. Scale bar = 5 μm. **B**. Representative images of dendritic spines of hippocampus after Golgi-Cox staining. Scale bar = 5 μm. **C**: Protrusion length. **D**: Average LWR. **E**: Percentage of immature filopodia-type spines. **F**: Percentage of mature mushroom spines. Results are mean ± S.E.M. (*n* = 14). Data of each individual animal is also presented. COR: cerebral cortex, HP: hippocampus, 3MA: CLP plus 3-MA, PDTC: CLP plus PDTC

Golgi staining showed that the length of spine protrusion was decreased in the cerebral cortex and hippocampus of mice with CLP. The average LWR of spines was increased by CLP. PDTC attenuated the decrease of spine protrusion length in the cerebral cortex and increase of average LWR in both cerebral cortex and hippocampus of mice with CLP. 3-MA reduces the increase of average LWR in the cerebral cortex of mice with CLP (Fig. 4A and D). These results suggest that CLP impairs spine formation and that this impairment is attenuated by PDTC and partially reduced by 3-MA.

CLP increased the percentage of filopodia in the hippocampus and decreased mushroom spines in the cerebral cortex and hippocampus. These changes are attenuated by PDTC. 3-MA also attenuated the increase of filopodia and decrease of mushroom in the hippocampus of mice with CLP (Fig. 4A, B, E and F). These results suggest that the maturity of spines is impaired by CLP and that the impairment is attenuated by PDTC and 3-MA.

**Fig. 5:**
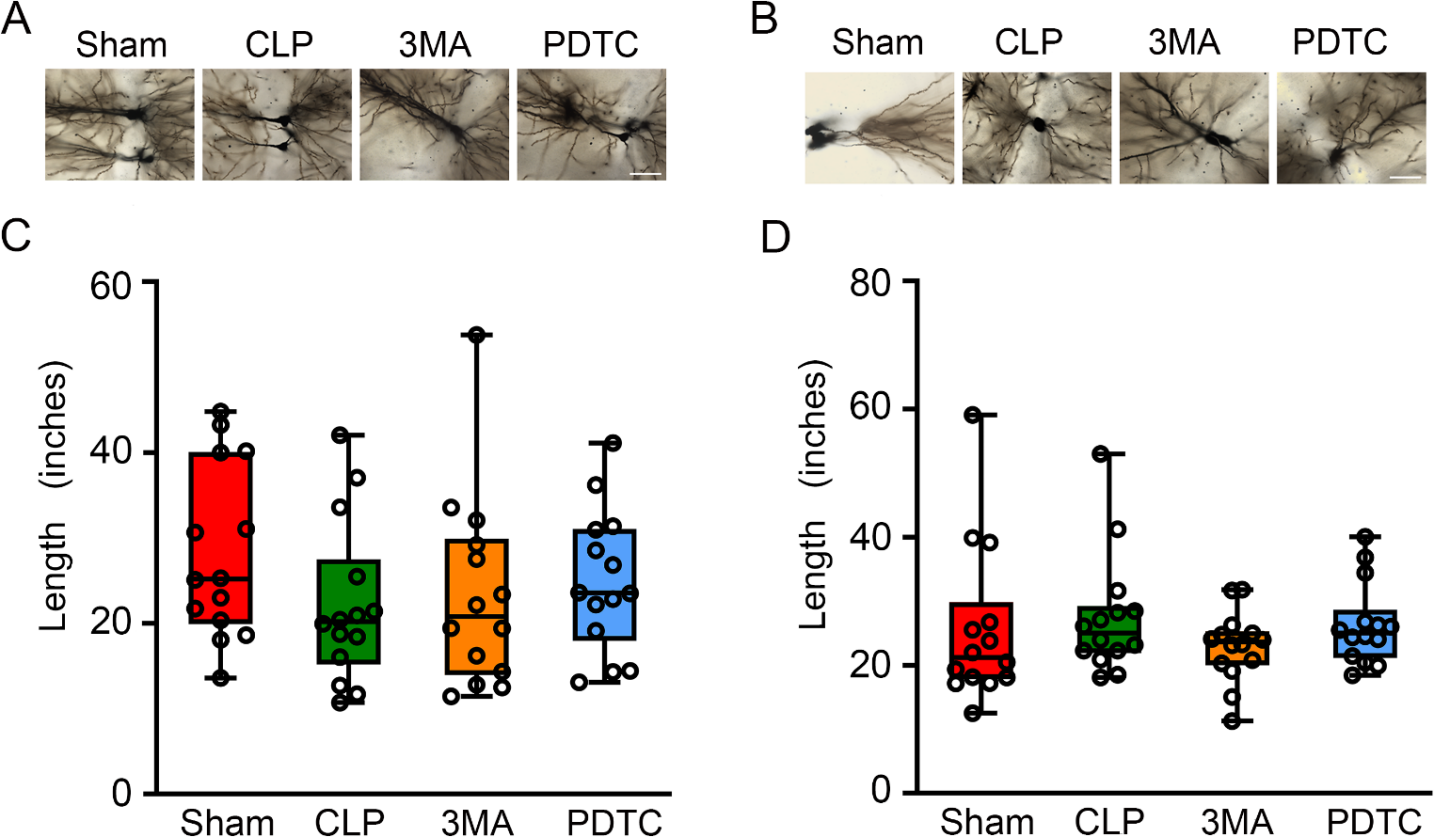
The effects of 3-MA and PDTC on dendritic length in mice with CLP. Cerebral cortex or hippocampus was harvested 7 days after CLP. **A**: Representative images of dendritic spines of cerebral cortex after Golgi-Cox staining. **B**. Representative images of dendritic spines of hippocampus after Golgi-Cox staining. Scale bar = 40 μm. **C**: Quantification of dendritic length in the cerebral cortex. **D**: Quantification of dendritic length in the hippocampus. Results are presented as median with interquartile range. Data of each individual animal is also presented (*n* = 14). COR: cerebral cortex, HP: hippocampus, 3MA: CLP plus 3-MA, PDTC: CLP plus PDTC

There were no differences in the total dendritic length of cerebral cortex and hippocampus among the 4 groups (Fig. 5), suggesting that CLP may not affect the macrostructure of dendritic trees.

### 3-MA and PDTC Attenuated CLP-Increased LC3II in the Microglia and Neurons of Brain Tissues

**Fig. 6:**
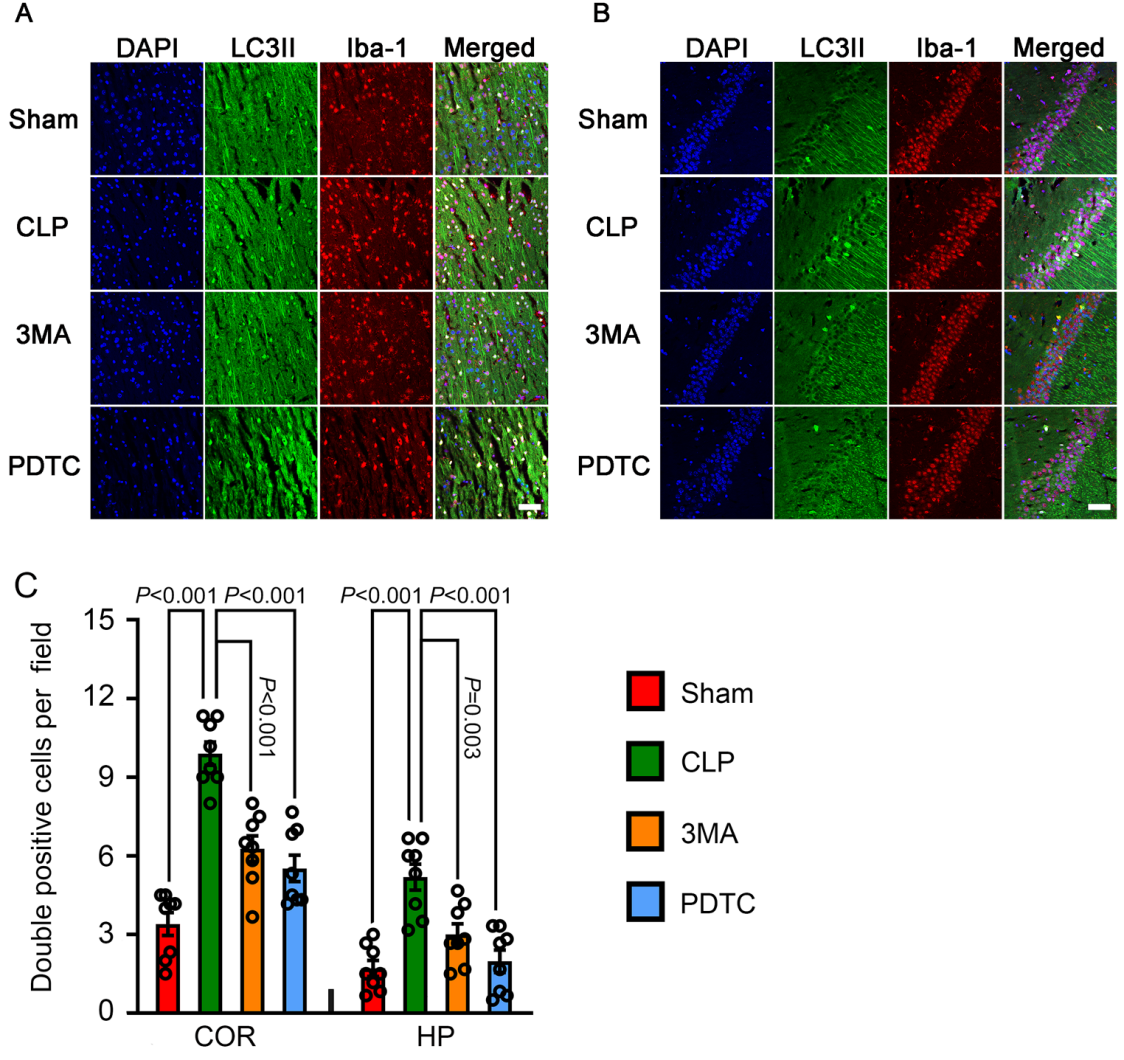
The effects of 3-MA and PDTC on number of cells positively stained for both LC3II and Iba-1 in mice with CLP. Brain tissues were harvested 24 h after CLP. **A**: Representative immunostaining images of cerebral cortex. **B**: Representative immunostaining images of hippocampus. **C**: Quantification of the number of cells positive for both LC3II and Iba-1. Scale bar = 50 μm. Results are mean ± S.E.M. (*n* = 8). Data of each individual animal is also presented. COR: cerebral cortex, HP: hippocampus, 3MA: CLP plus 3-MA, PDTC: CLP plus PDTC

**Fig. 7:**
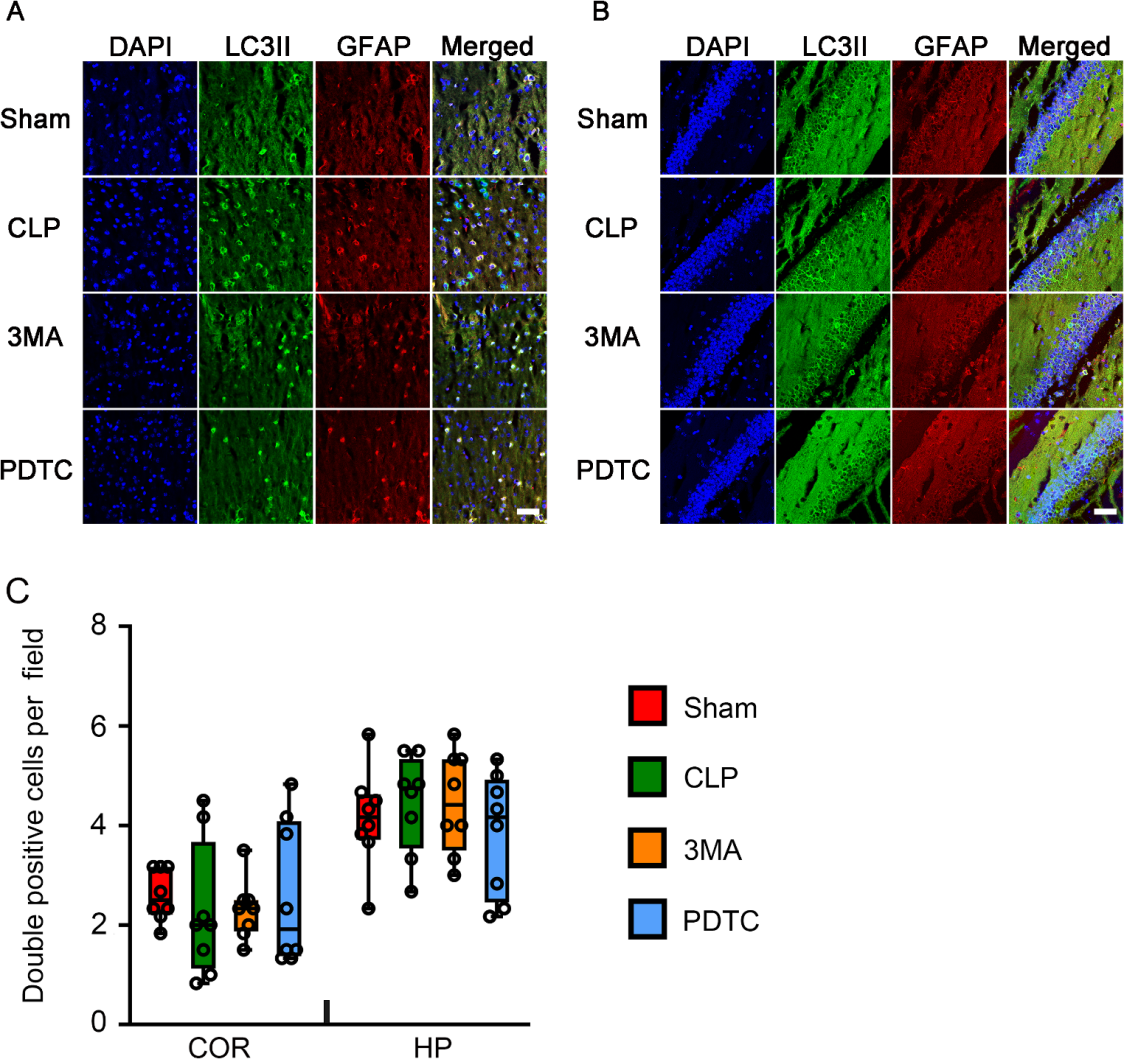
The effects of 3-MA and PDTC on number of cells positively stained for both LC3II and GFAP in mice with CLP. Brain tissues were harvested 24 h after CLP. **A**: Representative immunostaining images of cerebral cortex. **B**: Representative immunostaining images of hippocampus. **C**: Quantification of the number of cells positive for both LC3II and GFAP. Scale bar = 50 μm. Results are presented as median with interquartile range. Data of each individual animal is also presented (*n* = 8). COR: cerebral cortex, HP: hippocampus, 3MA: CLP plus 3-MA, PDTC: CLP plus PDTC

**Fig. 8:**
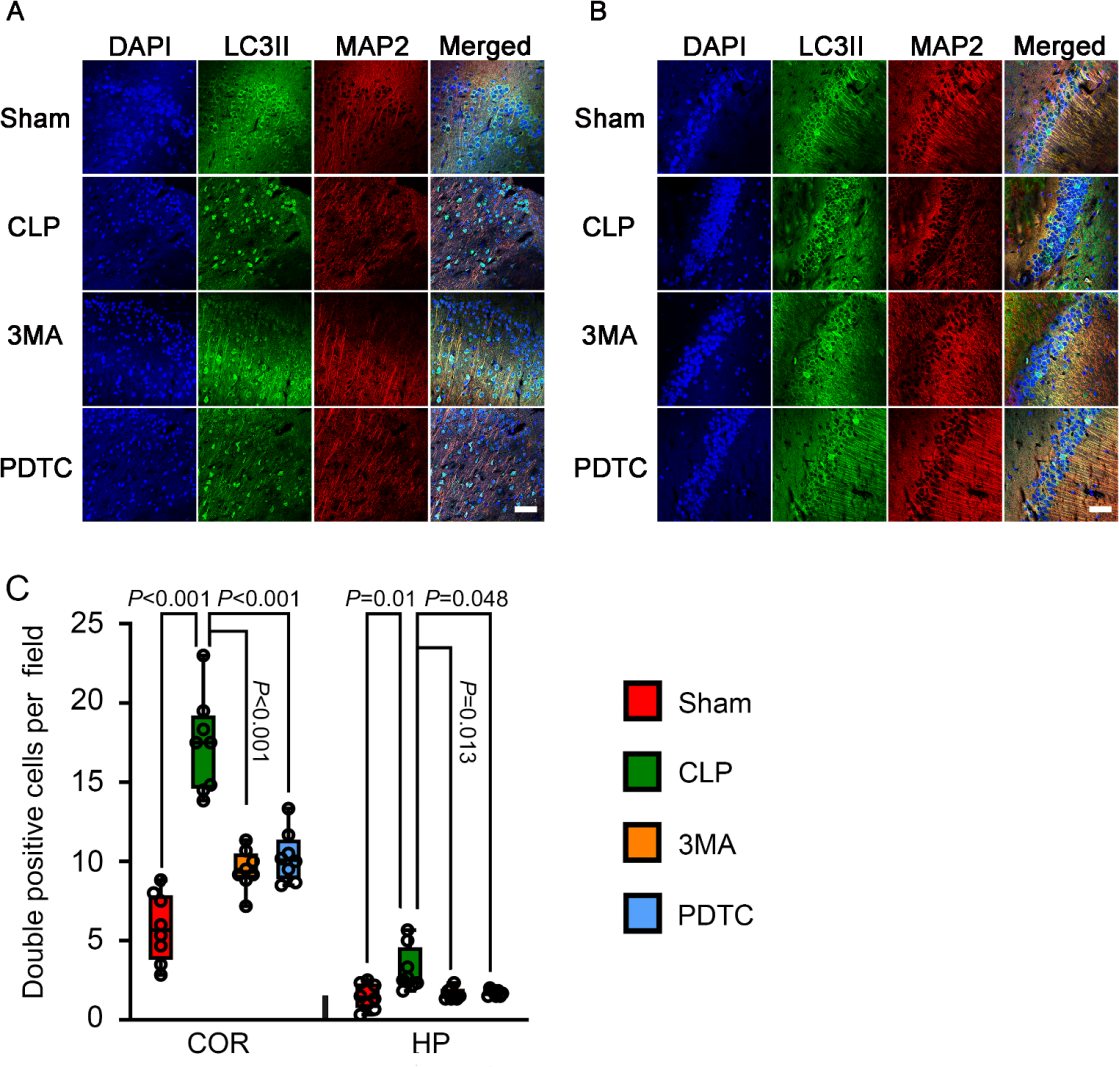
The effects of 3-MA and PDTC on number of cells positively stained for both LC3II and MAP2 in mice with CLP. Brain tissues were harvested 24 h after CLP. **A**: Representative immunostaining images of cerebral cortex. **B**: Representative immunostaining images of hippocampus. **C**: Quantification of the number of cells positive for both LC3II and MAP2. Scale bar = 50 μm. Results are presented as median with interquartile range. Data of each individual animal is also presented (*n* = 8). COR: cerebral cortex, HP: hippocampus, 3MA: CLP plus 3-MA, PDTC: CLP plus PDTC

To determine which types of brain cells had changes in autophagy in mice with CLP, co-immunostaining of brain sections was performed. CLP increased the number of cells that were positive for both LC3-II and Iba-1or LC3-II and MAP-2 but not the number of cells that were positive for LC3-II and GFAP in the cerebral cortex and hippocampus of mice with CLP. 3-MA and PDTC attenuated the increase in cells positively stained with LC3-II and Iba-1 or LC3-II and MAP-2 in mice with CLP (Figs. 6, 7 and 8). These results suggest that CLP increases autophagy in the microglia and neurons and that 3-MA and PDTC attenuate this increase. These results also suggest that CLP may not affect the autophagy in astrocytes.

## Discussion

Our results showed that there was an increase in autophagy in the brain of mice with sepsis. PDTC and 3-MA decreased autophagy and improved learning and memory in mice with sepsis. These results suggest that autophagy is a therapeutic target for reducing SAE.

Autophagy is known to be important in maintaining cellular homeostasis [12, 13]. However, excessive autophagy can be harmful [33, 34]. The core molecules of autophagy consist of many autophagy-associated gene (ATG) proteins, such as LC3 and p62/SQSTM1 that are essential to the formation of autophagic vacuoles in mammalian cells. During this process, LC3I in the cytoplasm is transformed into membrane-bound LC3II as an initial step to form autophagosome. p62/SQSTM1, the adaptor protein binding ubiquitinated LC3 in the autophagosome, is degraded during autophagy [35]. Thus, increased autophagy is reflected by high levels of LC3-II or a high ratio of LC3-II to LC3-I and decreased p62/SQSTM1 [36]. Of note, the increased numbers of autophagosomes do not necessarily suggest increased autophagy because blocked autophagosome-lysosome fusion can lead to the accumulation of autophagosomes. Since Beclin-1 facilitates autophagosome-lysosomal fusion [37], the expression of Beclin-1 was measured in our study. We found that the levels of LC3II and Beclin-1 were increased and that the level of p62/SQSTM1 was decreased in the brain tissues of septic mice. These results suggest that the autophagy in these tissues is increased. Consistent with these results, autophagosome was present in the electron microscopic images of mice with CLP. These findings are consistent with previous findings [19, 38]. Interestingly, our results suggest that there is an increase in autophagy in the microglia and neurons but not in the astrocytes of mice with CLP because the number of microglia and neurons expressing LC3-II was increased but the number of astrocytes expressing LC3-II was not changed in these mice. These findings indicate a novel idea that sepsis-increased autophagy may be cell type-specific.

Sepsis induces neuroinflammation. Consistent with this idea, TNFα and IL-6, proinflammatory cytokines [39, 40], were increased in the brain tissues of mice with CLP. However, there was no change in IL-1β, also a proinflammatory cytokine, and IL-10, an anti-inflammatory cytokine [40], in the brain tissues of mice with CLP compared with sham operated mice. This no change finding may be due to the lack of effect of CLP on IL-1β and IL-10 concentrations in the brain or the inappropriate time point at which the brain tissues were harvested.

Autophagy can regulate inflammatory responses via multiple mechanisms, such as affecting the health of inflammatory cells and the production of cytokines [21, 22]. Consistent with these possibilities, 3-MA reduced IL-6 and TNFα in the brain of mice with CLP. 3-MA also attenuated CLP-increased autophagy in the microglia, cells that participate in the inflammatory responses in the brain. Interestingly, PDTC, an anti-inflammatory agent [25, 26], reduced autophagy in the mice with CLP. These results provide initial evidence to suggest that inflammation increases autophagy in the brain of animals with sepsis. Thus, there may be a vicious cycle in the brain of mice with CLP: inflammation increases autophagy, and autophagy enhances inflammation. Consistent with this idea, inflammatory cytokines have been shown to increase autophagy (reviewed in [24]). The mutual enhancement between autophagy and neuroinflammation may be important for the development of SAE because both PDTC and 3-MA inhibited neuroinflammation, brain cell autophagy and injury, and dysfunction of learning and memory in mice with CLP.

It has been shown that promoting autophagy including the chaperone-mediated autophagy reduces learning and memory impairment in animals with sepsis [9, 11, 19]. Inhibiting astrocytic autophagy can aggravate learning and memory impairment [9]. However, our results suggest that inhibition of autophagy improves SAE-like cognitive impairment because CLP increased autophagy and inhibiting autophagy by 3-MA attenuated the learning and memory impairment in mice with CLP. Similar to our finding, a previous study has shown that knockdown of voltage dependent anion channel alleviates the cognitive impairment by decreasing autophagy [10]. The reasons for the different findings are not known. Different strains of mice, variations in the severity of sepsis and different methods used to alter autophagy may have contributed to the inconsistent findings among studies.

Of note, our results suggest a critical role of IL-6 and TNFα in the SAE-like behavioral changes because CLP increased IL-6 and TNFα and induced learning and memory impairment, and 3-MA and PDTC reduced IL-6 and TNFα and the impairment of learning and memory in mice with CLP. In supporting this role, a higher circulating IL-6 level correlates with worse cognitive function and steeper cognitive decline in the elderly patients [41]. Chronic exposure to IL-6 inhibits neurogenesis in the hippocampus of IL-6 transgenic mice [42]. Neuronal cluster size of cerebellar granule cell cultures and their synaptic proteins (synapsin I and II), enolase, and α-internexin are significantly decreased after IL-6 treatment [43]. TNFα is an important mediator for learning and memory impairment after surgery [44]. Thus, IL-6 and TNFα may be specific targets for reducing SAE-like behavior.

Dendritic spines undergo significant changes in morphology during learning and memory [45]. Immature spine forms, including long filopodia-type spines, are highly motile and are thought to initiate synaptic contact [46]. The fully mature mushroom spines are stable and have many neurotransmitter receptors to support a high synaptic activity [46, 47]. Our study showed that the spine formation and maturation were interrupted by CLP. Importantly, 3-MA and PDTC attenuated these impairments. These findings are consistent with the results of learning and memory, suggesting a role of increased autophagy in the impairment of brain structure and function of mice with CLP.

Our study has limitation. Obviously, one cannot extrapolate our findings in mice directly to humans. Only male mice were used in this initial study. Future studies will need to include female mice in the study to determine whether there is a gender difference in the sepsis-induced brain changes and the effects of 3-MA and PDTC on these changes. Finally, our study has shown that sepsis increases the autophagy in microglia and neurons. Future studies may be designed to determine the outcome of the increased autophagy in each of these two types of cells to investigate the contribution of autophagy in each cell type to brain function changes in SAE.

## Conclusions

Our study has shown that sepsis increases autophagy in the microglia and neurons of the mice with CLP. This increased autophagy may contribute to a vicious cycle between autophagy and neuroinflammation, which impairs dendritic spine formation and maturation, learning and memory. PDTC and 3-MA attenuate these SAE-like brain structural and functional changes.

## Data Availability

Data are available upon a reasonable request.

## Declarations

### Study Location

The whole study was performed in the University of Virginia.
